# Flash optimization of drug combinations for *Acinetobacter baumannii* with IDentif.AI-AMR

**DOI:** 10.1038/s44259-025-00079-2

**Published:** 2025-02-21

**Authors:** Kui You, Nurhidayah Binte Mohamed Yazid, Li Ming Chong, Lissa Hooi, Peter Wang, Isaiah Zhuang, Stephen Chua, Ethan Lim, Alrick Zi Xin Kok, Kalisvar Marimuthu, Shawn Vasoo, Oon Tek Ng, Conrad E. Z. Chan, Edward Kai-Hua Chow, Dean Ho

**Affiliations:** 1 Department of Biomedical Engineering, College of Design and Engineering, National University of Singapore, Singapore, Singapore; 2 Institute for Digital Medicine (WisDM), Yong Loo Lin School of Medicine, National University of Singapore, Singapore, Singapore; 3 The N.1 Institute for Health (N.1), National University of Singapore, Singapore, Singapore; 4 National Centre for Infectious Diseases (NCID), Singapore, Singapore; 5 Cancer Science Institute of Singapore, National University of Singapore, Singapore, Singapore; 6 Department of Pharmacology, Yong Loo Lin School of Medicine, National University of Singapore, Singapore, Singapore; 7 The Bia-Echo Asia Centre for Reproductive Longevity and Equality (ACRLE), National University of Singapore, Singapore, Singapore

**Keywords:** Biotechnology, Translational research

## Abstract

Antimicrobial resistance (AMR) is an emerging threat to global public health. Specifically, *Acinetobacter baumannii* (*A. baumannii*), one of the main pathogens driving the rise of nosocomial infections, is a Gram-negative bacillus that displays intrinsic resistance mechanisms and can also develop resistance by acquiring AMR genes from other bacteria. More importantly, it is resistant to nearly 90% of standard of care (SOC) antimicrobial treatments, resulting in unsatisfactory clinical outcomes and a high infection-associated mortality rate of over 30%. Currently, there is a growing challenge to sustainably develop novel antimicrobials in this ever-expanding arms race against AMR. Therefore, a sustainable workflow that properly manages healthcare resources to ultra-rapidly design optimal drug combinations for effective treatment is needed. In this study, the IDentif.AI-AMR platform was harnessed to pinpoint effective regimens against four *A. baumannii* clinical isolates from a pool of nine US FDA-approved drugs. Notably, IDentif.AI-pinpointed ampicillin-sulbactam/cefiderocol and cefiderocol/polymyxin B/rifampicin combinations were able to achieve 93.89 ± 5.95% and 92.23 ± 11.89% inhibition against the bacteria, respectively, and they may diversify the reservoir of treatment options for the indication. In addition, polymyxin B in combination with rifampicin exhibited broadly applicable efficacy and strong synergy across all tested clinical isolates, representing a potential treatment strategy for *A. baumannii*. IDentif.AI-pinpointed combinations may potentially serve as alternative treatment strategies for *A. baumannii*.

## Introduction

*Acinetobacter baumannii* (*A. baumannii*) is a Gram-negative bacterium and exists ubiquitously, and it is the leading cause of healthcare-associated infection globally. Notably, it can lead to a variety of nosocomial infections, such as pneumonia, bacteremia, and urinary tract infection^1^. The bacterium-associated infections may lead to mortality rates of up to 30%^2^. Furthermore, multi-drug resistance (MDR) *A. baumannii* strains are resistant to multiple classes of antibiotics including carbapenems, which are considered the last-resort antibiotics against multi-drug resistant (MDR) Gram-negative pathogens. Typically, MDR is geographically specific. For example, a recent study that surveyed 453 hospitals in 48 countries reported that *A. baumannii* has a high chance of being multi-drug resistance^3^. The MDR rates in Europe may exceed 93%, while North America has the lowest rates of 47%. Consequently, widespread resistance to antibiotics within the *A. baumannii* family results in unsatisfactory clinical outcomes. Moreover, this pathogen has been classified by the World Health Organization (WHO) as one of the “ESKAPE” pathogens, a list of highly virulent and resistant pathogens^4^. On May 17, 2024, WHO published a bacterial priority pathogens list categorizing *A. baumannii* as a pathogen of critical priority, further suggesting that there is an urgent need to develop treatment strategies for this pathogen^5^.

Carbapenems have conventionally played a critical role in *A. baumannii* treatment strategies. The emergence of carbapenem- and multi-drug resistant strains has substantially exhausted treatment options for the pathogen^6^. To further explore other strategies, combination therapies have been prioritized for treating this bacterial infection^6^. However, combinatorial designs have not been developed or well-studied specifically for *A. baumannii*. Aside from carbapenems, colistin is also prescribed as a last-resort defense against MDR gram-negative bacteria^7^, and it is sometimes paired with other drugs including carbapenems, serving as the backbone of combination therapies^6,8^. However, multiple studies including a clinical trial (NCT01597973) have reported that colistin monotherapy is not superior to colistin-based combination therapies in the context of clinical outcomes^7–9^. These outcomes can be attributed to the absence of drug-dose optimization. For instance, previous studies have reported that properly pairing drugs in combinations and determining the optimal dosage ratios are critical to improved clinical outcomes^10–12^. Therefore, it is important to specifically design drug combinations for *A. baumannii*.

Conventional approaches including high throughput screening, higher order drug development, and synergy prediction have been deployed to design combination therapies for various disease indications^13–18^. Although there was a rapid increase in FDA-approved antimicrobial drugs per year from 2013 to 2019 due to economic incentives, the majority of these drugs do not meet the critical need to treat the “ESKAPE” pathogens^19,20^. In addition, several of these drugs, either approved or in development, were found to be redundant as they target the same resistant pathogen^19^. Despite the significant amount of funding and resources invested into drug development, there are currently 43 antibiotics in development with 15 in phase 1 clinical trials, 13 in phase 2, and 13 in phase 3. Of these candidates, a high attrition rate was observed and roughly 15 antimicrobial drugs in development have the potential to treat “ESKAPE” pathogens of critical threats, such as *Acinetobacter baumannii* and *Pseudomonas aeruginosa*^20^. This presents a challenge to sustainable efficacy-driven drug development.

As part of the strategy to overcome this challenge, the emergence of AI has also led to accelerated pathways towards sustainable drug development and discovery^13,21–23^. However, optimizing effective drug combinations while determining their optimal dose ratios is a challenging task in the context of drug development. For example, determining the top combinations from a pool of nine drugs at three concentration levels would need to experimentally assess 19,683 combinations (3^9^) in the parameter space, which is unsustainable. To properly manage healthcare resources and overcome the aforementioned challenges, IDentif.AI-AMR was developed as a sustainable approach to ultra-rapidly pinpoint top combinations against *A. baumannii* using a small amount of prospectively obtained data. IDentif.AI harnesses a second-order quadratic series that correlates the drug combinations and their corresponding efficacy from the small dataset^24–28^. This relationship was originally discovered via neural networks and has since been utilized for various indications spanning from in vitro to human studies^12,24,25,27–35^. More importantly, IDentif.AI does not involve pre-existing or big data to formulate the above-mentioned equation, and the platform can rapidly pinpoint top combinations in the parameter space using the second-order equation^26–28^.

**Fig. 1:**
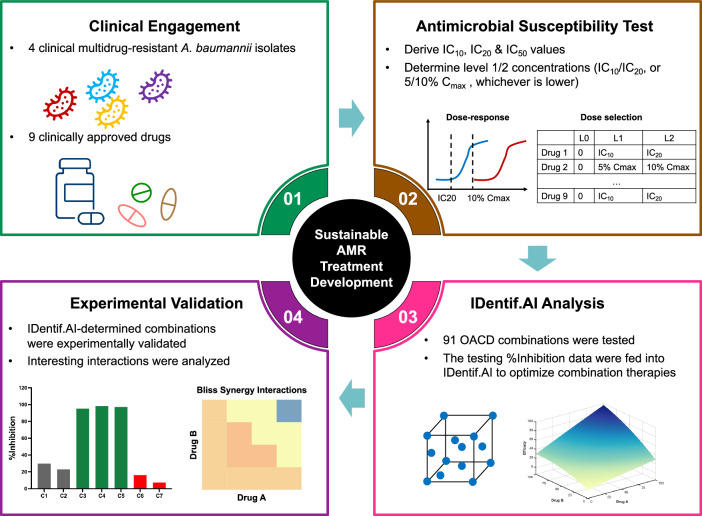
Sustainable AMR treatment development workflow. The IDentif.AI workflow begins with engaging with clinical teams to shortlist the initial pool of drugs for IDentif.AI analysis. Subsequently, IDentif.AI harnesses the experimental data derived from a small set of experiments to rapidly pinpoint effective combinations against the bacteria. IDentif.AI-pinpointed top combinations are experimentally validated. This workflow may sustainably accelerate the development of *A. baumannii* and other AMR-specific treatment strategies.

In this study, the initial pool of nine FDA-approved drugs was shortlisted by engaging with infectious disease experts from the National Centre for Infectious Diseases (NCID) in Singapore. These drugs including meropenem (MEM), tigecycline (TGC), polymyxin B (PB), minocycline (MI), amikacin (AN), ampicillin-sulbactam (SAM), rifampicin (RA), eravacycline (ERV), and cefiderocol (FDC) were selected to determine top combinations against extremely drug-resistant (XDR) *A. baumannii* {2023496441} using IDentif.AI (Fig. 1). A set of 91 strategic combinations were experimentally validated, and IDentif.AI harnessed these combinations to interrogate the interaction space among nine drugs. Top combinations were experimentally validated. Notably, IDentif.AI-pinpointed SAM/FDC, SAM/FDC/RA, and FDC/PB/RA combinations exhibited 93.89 ± 5.95%, 96.63 ± 4.87%, and 92.23 ± 11.89% inhibition against the bacteria, respectively. Moreover, IDentif.AI determined PB/RA combination may exhibit strong interactions. Subsequently, this combination was comprehensively assessed in three additional clinical isolates, and the results suggested that the PB/RA combination has strong synergy and may have broadly applicable efficacy. These combinations were ultra-rapidly pinpointed and assessed via the IDentif.AI workflow in less than two weeks (Fig. 1), representing a sustainable approach to accelerate AMR-specific drug development. IDentif.AI-pinpointed combinations may therefore serve as sustainable alternative treatment strategies for XDR *A. baumannii* and diversify the options available for clinical teams. The overarching theme of this study is demonstrating a sustainable and community-driven workflow that may be deployed to properly design drug combinations in the context of pandemic readiness, regionally and internationally.

## Results

### *A. baumannii* antimicrobial resistance to single-drug treatment

The initial pool of drugs was selected by engaging with clinicians and infectious disease experts from the National Centre for Infectious Diseases (NCID) in Singapore, and the selected drugs were clinically relevant, available locally, and combinatorial treatment potentially feasible based on their known side effect profiles and previously published data on general activity against *A. baumanii* (Supplementary Table 1). The antimicrobial effects of the nine drugs were experimentally assessed via antimicrobial susceptibility testing. First, XDR *A. baumannii* {2023496441} was exposed to the drugs individually in 12 different concentrations (2-fold serial dilutions) at 35 °C for 20 h without shaking (Supplementary Table 2). The bacterial growth was determined via optical density (OD) at 600 nm (*n* = 3). To construct the dose-response curves, the logarithmic scale of drug concentrations was plotted against the measured %Inhibitions for each drug (Supplementary Fig. 1). The IC_10_, IC_20,_ and IC_50_ values for each drug were extracted from the curves, and most of the drugs had no activity against the bacteria (Supplementary Fig. 1), which aligns with the intrinsic resistance exhibited by *A. baumannii* {2023496441}. The overall Z’-factor for dose-response experiment was 0.599, indicating an “excellent assay.”

### Selecting drug concentrations for IDentif.AI analysis

The parameter space interrogated by IDentif.AI consisted of nine drugs in three different concentration levels: L0, L1, and L2. L0 represents zero concentration while L1 and L2 concentrations are selected based on both dose-response analysis (IC_10_ and IC_20_) and pharmacological data (*C*_max_; maximum serum concentration that a drug can achieve). Aside from ensuring the clinical feasibility of the selected drugs, the concentrations selected for drug combinatorial design must also be clinically relevant. In general, 10% of *C*_max_ is considered as the drug concentration achievable at the targeted site in human^12^. As such, L1 and L2 are selected based on either IC_10_/IC_20_ or 5/10% of *C*_max_. The highest L2 concentrations are limited to IC_20_ to ensure that no single drug is overpowering combinatorial designs, enabling IDentif.AI to detect drug-drug interactions. The selected concentration levels for each of the nine drugs were evaluated against *A. baumannii* (Table 1 and Supplementary Fig. 2). Across all nine drugs, the L1 and L2 concentrations mostly resulted in no efficacy against *A. baumannii* {2023496441} (*n* = 3).

**Table 1:** Selected drug concentrations for IDentif.AI analysis

Drug	IC_50_ (μg/mL)	Maximum Plasma Concentration (*C*_max_)		IDentif.AI analysis	
		Conc. (μg/mL)	Dosing Scheme	Level 1 (μg/mL)	Level 2 (μg/mL)
Amikacin	>100	2.8^75^	After 3 months of once-daily inhalation of 590 mg amikacin	0.14	0.28
Ampicilin-sulbactam	50.770	NA	NA	32.47	37.54
Cefiderocol	1.286	91.4^76^	A single 3 h IV dose of 2 g cefiderocol	1.126	1.196
Eravacycline	>5	2.125^77^	A single IV dose of 1 mg/kg eravacycline every 12 h	0.106	0.213
Meropenem	51.610	49^78^	A single IV dose of 1 g meropenem for 30 min	2.45	4.9
Minocycline	>2	2.92^79^	An oral dose of 135 mg per day minocycline	0.146	0.292
Polymyxin B	1.592	5.53^80^	After a loading dose of 2.5 mg/kg Polymyxin B for 3 h infusion	0.277	0.553
Rifampicin	1.667	7^81^	An oral single dose of 600 mg rifampicin	0.35	0.7
Tigecycline	>5	1.45^82^	A single IV dose of 100 mg tigecycline	0.0725	0.145

The L1 and L2 concentrations for IDentif.AI analysis were selected based on either IC_10_/IC_20_ or 5/10% of *C*_max_.

### Pinpointing effective drug combinations using IDentif.AI

A list of 91 carefully designed orthogonal array composite design (OACD) combinations consisting of various drugs in different concentration levels (L0, L1, L2) was prepared (Supplementary Table 3). This OACD design is comprised of 64 two-level combinations derived from fractional factorials and 27 three-level combinations generated via orthogonal arrays. The bacterial cultures (35 °C for 20 h without shaking) of *A. baumannii* {2023496441} were then exposed to all 91 combinations (*n* = 3), and the growth profile (%Inhibition) was determined via MIC assay (OD_600_) (*n* = 3). Subsequently, IDentif.AI correlated the %Inhibition data of all 91 OACD combinations and the monotherapies of all nine drugs (L1 and L2) (*n* = 3) via a second order quadratic series, which was used to describe the relationship between input combinations and their corresponding efficacies (Supplementary Table 4). No transformation was applied to the %Inhibition data as suggested by Box-Cox transformation, and no outliers were identified via residual-based outlier analysis (Supplementary Fig. 3). IDentif.AI analysis resulted in an adjusted R^2^ of 0.767, pointing to a highly correlated relationship between input combinations and output %Inhibitions (goodness-of-fit) (Supplementary Table 4). The Z’-factor for IDentif.AI analysis was 0.746, suggesting an “excellent assay.”

**Fig. 2:**
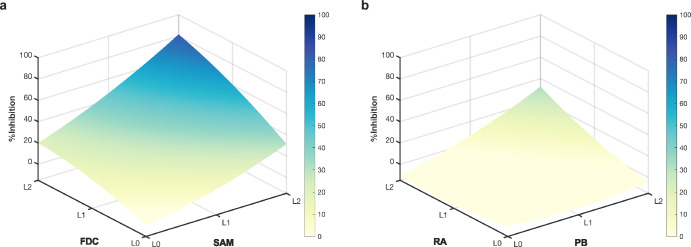
IDentif.AI-predicted drug interactions. IDentif.AI pointed to interesting drug-drug interactions in SAM/FDC and PB/RA combinations. **a** SAM and FDC may interact at L2 concentrations to enhance %Inhibition against the bacteria. **b** Similarly, PB/RA may exhibit strong interactions when drug concentrations achieve L2. Ampicillin-sulbactam (SAM), cefiderocol (FDC), polymyxin B (PB), and rifampicin (RA).

A ranked list of all possible combinations in the parameter space (3^9^ = 19,683) and their predicted inhibitions was provided based on IDentif.AI-estimated coefficients (second order quadratic series) (Supplementary Table 4). However, considering therapeutic compliance and adherence, only combinations consisting of four or less drugs were prioritized (Table 2)^27,36,37^. To further validate the actionability of IDentif.AI in accelerating combinatorial design, three low-ranked combinations that may result in near zero %Inhibition efficacy against the bacteria were also selected (Table 2). Additionally, IDentif.AI also detected potential drug-drug interactions in two combinations: SAM/FDC and PB/RA (Fig. 2). Based on the prediction, IDentif.AI determined that both combinations may exhibit synergy when drugs interact at higher concentrations. Further examining the shortlisted combinations in Table 2, multiple 3- and 4-drug combinations are comprised of SAM/FDC and PB/RA, suggesting that they may serve as the backbone of higher order drug combinations. Similarly, IDentif.AI-pinpointed 3-drug combinations are also a part of 4-drug combinations (e.g. SAM/FDC/TGC and SAM/FDC/TGC/MI). More importantly, 3- and 4-drug combinations may diversify treatment targets, and they are less likely for the bacteria strain to simultaneously develop resistance across different pathways or targets^38^. In the context of clinical feasibility, these 3- and 4-drug combinations should be prioritized. However, 5-drug and higher-order combinations were not shortlisted for validation as multi-drug regimens could potentially reduce patient compliance^27,37^. They become increasingly complex and may cause incorrect usage or missed doses, which may compromise patients’ treatment and further increase the rates of antimicrobial resistance. Importantly, with 4-drug combinations such as SAM/FDC/TGC/MI conferring nearly 100%Inhibition, it is unnecessary to introduce additional drugs to the regimen. Besides, 2-, 3-, and 4-drug combinations may improve patient compliance and treatment outcomes. Further investigations pertaining to the above-mentioned combinations are needed to quantify and evaluate their interactions. Combinations of interest were shortlisted for experimental validation (Table 2).

**Table 2:** Predicted and prospectively measured %Inhibitions of IDentif.AI-pinpointed combinations

Drug Combinations	Rank	Predicted %Inhibition	Measured %Inhibition
*Top 2-drug Combinations*			
SAM(37.540) + FDC(1.196)	8535	78.05	93.89 ± 5.95
FDC(1.196) + TGC(0.145)	13,188	59.97	31.49 ± 10.63
PB(0.553) + RA(0.700)	18,438	29.08	81.87 ± 31.37
*Top 3-drug Combinations*			
SAM(37.54) + FDC(1.196) + TGC(0.145)	1992	104.46	86.92 ± 17.77
SAM(37.54) + FDC(1.196) + RA(0.700)	7793	80.82	96.63 ± 4.87
FDC(1.196) + PB(0.553) + RA(0.700)	9312	75.23	92.23 ± 11.89
*Top 4-drug Combinations*			
SAM(37.540) + FDC(1.196) + TGC(0.145) + MI(0.146)	429	118.23	99.27 ± 0.89
SAM(37.540) + FDC(1.196) + AN(0.140) + TGC(0.145)	758	113.66	96.00 ± 4.74
FDC(1.196) + MI(0.292) + PB(0.553) + RA(0.700)	2022	107.72	91.88 ± 6.43
*Ineffective Combinations*			
RA(0.350) + AN(0.280)	19,666	−12.80	−21.00 ± 15.37
PB(0.277) + ERV(0.106)	19,674	−16.93	−17.21 ± 15.18
AN(0.140) + ERV(0.106)	19,679	−17.79	3.09 ± 13.28
*Monotherapies*			
SAM(37.540)			50.70 ± 18.43
FDC(1.196)			4.78 ± 10.48
TGC(0.145)			23.19 ± 10.47
PB(0.227)			−2.60 ± 12.85
PB(0.553)			0.05 ± 17.10
RA(0.350)			−1.47 ± 8.60
RA(0.700)			6.27 ± 10.76
MI(0.146)			−4.59 ± 9.74
MI(0.292)			−9.40 ± 7.84
AN(0.140)			−5.91 ± 9.51
AN(0.280)			−6.42 ± 9.63
ERV(0.106)			−3.59 ± 8.75

Prioritized top 2-, 3-, 4-drug, and low-ranked combinations and respective monotherapies were evaluated experimentally (*n* = 3). The predicted %Inhibitions are also provided along with the rank within the ranked list of 19,683 combinations. *MEM* Meropenem, *TGC* tigecycline, *PB* polymyxin B, *MI* minocycline, *AN* amikacin, *SAM* ampicillin-sulbactam, *RA* rifampicin, *ERV* eravacycline, *FDC* cefiderocol.

### Experimental validation of IDentif.AI-pinpointed combinations

In the validation study, *A. baumannii* {2023496441} (35 °C for 20 h without shaking) was exposed to shortlisted top and low-ranked IDentif.AI-pinpointed combinations. Overall, IDentif.AI-pinpointed combinations were able to achieve %Inhibition greater than 80 (Table 2). For instance, PB (0.553 μg/mL) and RA (0.700 μg/mL) monotherapies exhibited 0.05 ± 17.10% and 6.27 ± 10.76% inhibition (*n* = 3), respectively, and when paired in combination, PB/RA was able to inhibit 81.87 ± 31.37% of the growth of *A. baumannii* (*n* = 3) (Table 2). The interaction between PB and RA is in line with the IDentif.AI analysis in Fig. 2b. Furthermore, pairing PB/RA with FDC in a 3-drug combination, FDC/PB/RA was able to inhibit 92.23 ± 11.89% (*n* = 3) of the bacteria (Table 2). Similarly, in a 4-drug combination comprising of FDC/PB/RA and an additional drug (MI), FDC/MI/PB/RA combination exhibited 91.88 ± 6.43%Inhibition efficacy (*n* = 3). Furthermore, SAM (37.540 μg/mL) and FDC (1.196 μg/mL) monotherapies resulted in 50.70 ± 18.43 and 4.78 ± 10.48%Inhibition, respectively (*n* = 3). However, when paired in combination, SAM/FDC inhibited 93.89 ± 5.95% of the bacteria (*n* = 3) (Table 2). This result is also consistent with the predicted interaction in Fig. 2a. Pairing SAM/FDC with RA in a 3-drug combination also resulted in substantially improved efficacy (96.63 ± 4.87%Inhibition). Importantly, most of top 4-drug combinations are comprised of SAM/FDC. For example, SAM/FDC/TFC/MI combination achieved 99.27 ± 0.89% Inhibition against the bacteria (*n* = 3). These results suggest that the PB/RA and SAM/FDC combinations may potentially serve as the backbone of combinatorial design specifically for *A. baumannii* treatment.

To evaluate the versatility of the IDentif.AI platform, three low-ranked combinations were experimentally validated. Notably, RA (0.350 μg/mL) in combination with AN (0.280 μg/mL) resulted in −21 ± 15.37%Inhibition against the bacteria (*n* = 3). In addition, pairing PB (0.277 μg/mL) with ERV (0.106 μg/mL) inhibited −17.21 ± 15.18% of the bacteria (*n* = 3) (Table 2). In sum, these two combinations had no effects against *A. baumannii*. Moreover, it is important to realize that completely different results were obtained from simply replacing a drug with the efficacious PB/RA combination. Thus, pairing the correct drugs in combination is essential to achieve optimal outcomes. The inefficacy of the low-ranked combinations along with other factors including well-to-well subtle differences (e.g. temperature and oxygen level) may have led to the negative %Inhibition values. Additionally, the hormetic effect, which is described as the adaptive response of bacteria to stimulations, may potentially contribute to the observed negative %Inhibition values^39^.

The experimentally derived %Inhibition data of IDentif.AI-pinpointed top and low-ranked combinations were further analyzed with their respective predicted efficacy. The Pearson correlation coefficient (*r*) of all validated combinations in Table 2 was 0.89, suggesting a strong correlation between IDentif.AI-predicted and measured %Inhibition (Supplementary Fig. 4a). The residual plot of these combinations was also plotted in Supplementary Fig. 4b. Moreover, the %Inhibition efficacy of PB/RA was statistically significant when compared to the low-ranked RA/AN combination (Supplementary Fig. 4c). The Z’-factor for the validation study was 0.655, indicating an “excellent assay.”

### Synergy analysis for SAM/FDC and PB/RA

**Fig. 3:**
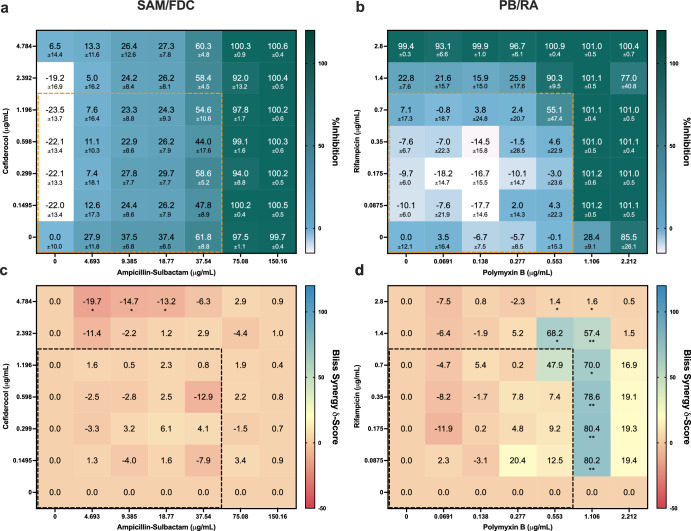
Synergy analysis of SAM/FDC and PB/RA combinations in *A. baumannii* {2023496441}. **a** The interaction and **c** synergy maps of SAM/FDC indicated that the combination was mostly driven by SAM with no observed synergistic interactions. **b** The interaction map demonstrated that PB/RA combination exhibited strong interactions at higher concentrations. **d** The synergistic interactions of PB/RA were further confirmed in the synergy map. When PB was administered at 1.106 *μ*g/mL, the combination achieved synergy scores of >50 across multiple dose ratios. The dotted boxes represent the concentration ranges within the original L2 concentrations. Bliss δ-synergy scores < 10, > 10, and in between −10 and 10 represent antagonistic, synergistic, and additive interactions. The significance of Bliss δ-synergy scores was assessed using one-sample *t*-test (*P < 0.05, **P < 0.01 and ***P < 0.001). Data are presented in mean ± propagated SD (*n* = 3).

SAM/FDC and PB/RA combinations were initially detected by IDentif.AI to potentially exhibit interesting interactions. Subsequent validation results in Table 2 also suggested that these two combinations may interact at higher concentrations. To further evaluate their drug-drug interactions in *A. baumannii* {2023496441}, a 7 × 7 checkerboard analysis was performed to obtain the %Inhibitions of various dose ratios for both combinations. The highest tested concentrations were based on 4× of the original L2 concentrations (2-fold dilutions). Bliss independence model synergy analysis was performed based on the %Inhibition data, and synergy scores < 10, > 10, and between −10 and 10 are considered antagonistic, synergistic, and additive interactions, respectively (Fig. 3)^26–28,40,41^. The interaction map of SAM/FDC indicated that the drug-drug interactions were mostly driven by SAM as its monotherapies were relatively more potent (Fig. 3a). Further examining the Bliss synergy map indicated that across all dose ratios in the checkerboard, the combination did not exhibit any significant synergistic interactions against the bacteria (Fig. 3c). Even though the validation results in Table 2 pointed to some interactions between SAM and FDC at L2 concentrations, no dose ratios exhibited synergistic interactions across the checkerboard (Fig. 3c). More importantly, SAM/FDC combination exhibited significant antagonistic interactions when FDC is administered at 4.784 μg/mL (Fig. 3c). Thus, this combination was not further evaluated in additional clinical isolates.

In Fig. 3b, the interaction map of PB/RA pointed to potential strong interactions at various dose ratios. This result aligned with multiple studies that have pointed to synergistic interactions between PB and RA^42–44^. The PB/RA combination may have strong interactions when PB is dosed at 1.106 μg/mL while RA is administered at low doses. For example, PB (1.106 μg/mL) and RA (0.0875 μg/mL) exhibited 28.4 ± 9.1 and −10.1 ± 6.0%Inhibitions, respectively, and when paired in combination, PB/RA inhibited 101.2 ± 0.5% of the bacteria (Fig. 3b). Similar strong interactions were also observed at other dose ratios when PB is at 1.106 μg/mL. To quantify the drug-drug interactions, Bliss synergy analysis was performed based on the 7 × 7 checkerboard. PB (1.106 μg/mL) in combination with RA (0.700 μg/mL) resulted in a synergy score of 80.2, indicating a strong synergistic interaction (Fig. 3d). Overall, the analysis suggested that PB/RA exhibited stronger synergistic interactions at lower concentrations, especially when PB is at 1.106 μg/mL (Fig. 3d). As a result, PB/RA was further evaluated in additional clinical isolates. In sum, properly pairing these two drugs in combinations and pinpointing their optimal dose ratios are essential to achieve the best outcomes.

### Broadly applicable activity in additional clinical isolates

**Fig. 4:**
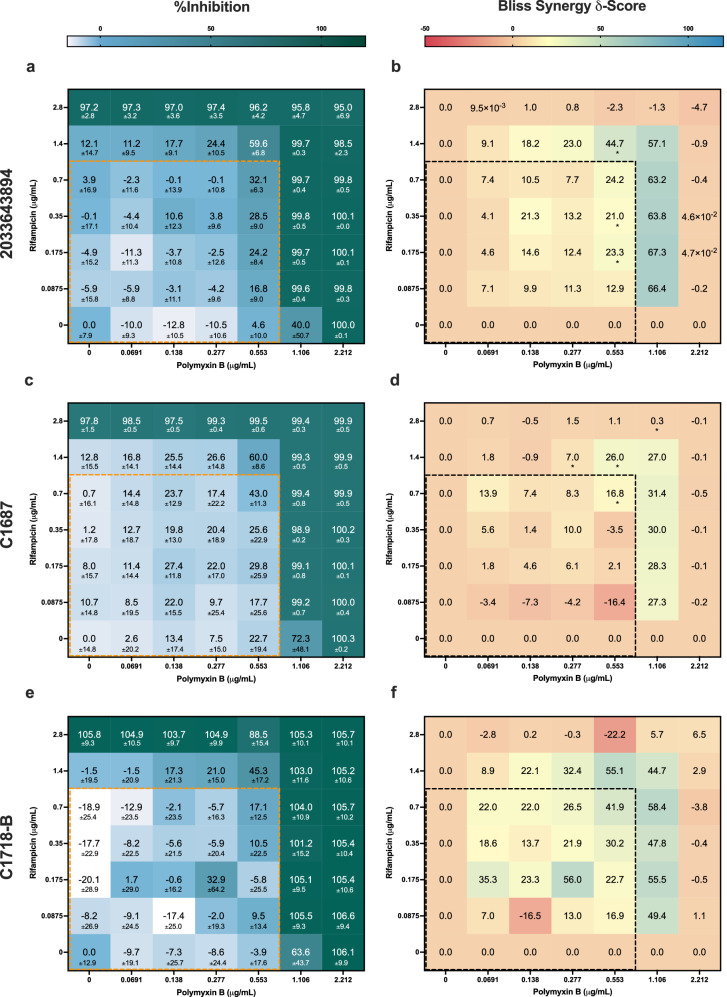
Determining the broad applicability of PB/RA combination in additional *A. baumannii* clinical isolates. PB/RA combination was further assessed in a 7 × 7 checkerboard in three additional clinical isolates: **a** 2033643894, **c** C1687, and **e** C1718-B. The synergy maps of **b** 2033643894 and **d** C1687 demonstrated that PB/RA exhibited stronger synergistic interactions outside of the originally tested L2 concentrations. In contrast, **f** PB/RA synergistically interacted at lower concentrations. The PB/RA combination demonstrated broadly applicable efficacy across all tested clinical isolates. The dotted boxes represent the concentration ranges within the original L2 concentrations. Bliss δ-synergy scores < 10, > 10, and in between −10 and 10 represent antagonistic, synergistic, and additive interactions. The significance of Bliss δ-synergy scores was assessed using one-sample *t*-test (*P < 0.05, **P < 0.01, and ***P < 0.001). Data are presented in mean ± propagated SD (*n* = 3).

PB in combination with RA exhibited strong synergistic interactions in *A. baumannii* {2023496441} when assessed via Bliss independence model. Thus, to further evaluate its applicability in other strains, PB/RA was assessed in three additional pan-resistant *A. baumannii* clinical isolates: 2033643894, C1687, and C1718-B (Supplementary Tables 1 and 2). These strains were selected as they exhibit the broadest resistance profiles, and they would potentially be challenging infections to treat. Similarly, each of the isolates was exposed to PB/RA in a 7 × 7 checkerboard assay (2x of L2 concentrations). The %Inhibition results of the checkerboards were evaluated using Bliss synergy analysis. In Fig. 4, PB/RA exhibited strong synergistic interactions across all three tested clinical isolates. Notably, the combination exhibited strong interactions at higher concentrations for both 2033643894 and C1687 isolates (Fig. 4a, b). However, for C1718-B isolates, PB/RA exhibited stronger interactions at lower concentrations (<L2 concentrations) (Fig. 4c). Though PB/RA exhibited broadly applicable efficacy across multiple clinical isolates, it is important to note that due to dose-dependent synergy, pinpointing the optimal dose ratios for each isolate (and those beyond the tested isolates) may lead to be best outcomes.

## Discussion

### Sustainable drug combination optimization using IDentif.AI-AMR

Under SOC, AMR is often tested with the AST on a positive culture isolated either from the patient’s site of infection, urine, or blood culture^45^. The AST enables clinical microbiologists and infectious disease physicians to pinpoint an effective antimicrobial regimen for individual patients. The clinical MIC obtained typically categorizes the isolate’s response to these monotherapies under susceptible (S), intermediate (I), or resistant (R). Even though current rapid AST has the capability to provide results within 8 h or less^45^, the drug combinations used in these tests are often determined empirically via monotherapies^43^. Moreover, these tests do not guide clinical microbiologists and infectious disease physicians in optimizing drug combinations and doses. The profound effects of the intricate drug interactions (Fig. 3) within the culture pose a logistical challenge and is unsustainable to screen all the possible drug combinations (3^9^ = 19,683). Conventionally, AST-driven drug combinations do not consider therapeutics that may be resistant to the bacteria (e.g. high IC_50_). For example, SAM has an IC_50_ of 50.770 μg/mL (Table 1), which typically indicates the bacteria is resistant (R) to the drug. However, IDentif.AI included it in the initial pool of drugs and later prioritized SAM in combination with FDC as one of the top combinations to effectively inhibit *A. baumannii*.

In this study, harnessing only the data of 91 combinations, IDentif.AI was able to ultra-rapidly determine top drug combinations and doses (Table 2). This rapid and sustainable workflow may enhance the current AST-driven approach with better healthcare resource management. In the long run, the reduced number of combinations minimizes the amount of logistics and resources required to accelerate AMR drug development^46,47^. For instance, in less than two weeks, IDentif.AI was able to sustainably pinpoint the PB/RA combination, which exhibited broadly applicable efficacy across four *A. baumannii* clinical isolates, and the optimal dose ratios for each strain were also pinpointed (Figs. 3b and 4). However, it is important to note that PB/RA combination including its mechanism has been well studied in previous works^42,43^. An in vitro study that evaluated PB/RA against various multi-drug resistance *A. baumannii clinical isolates* in the United Kingdom concluded that the efficacy of the combination is dependent on the strains and does not support the empirical use of drug combinations^42^. Similar to the drug-drug interactions observed in Figs. 3 and 4, the optimal dose ratios of PB/RA, especially where synergistic interactions are observed, substantially different within the tested strains. For example, PB/RA exhibited stronger synergy at higher concentrations in C1687 strain, while the combination exhibited strong synergy at lower concentrations in C1718-B. In a series of studies by Zhao et al. from Monash University in Australia, PB/RA combination was comprehensively investigated in both in vitro and in vivo studies^44^. The synergy of PB/RA combination was pinpointed at 0.75 μg/mL/1.00 μg/mL (0.75 dose ratio) and 0.50 μg/mL/2.00 μg/mL (0.25 dose ratio). Though these same exact dose ratios were not tested, PB/RA in similar dose ratio (PB 1.106 μg/mL/RA 1.400 μg/mL; 0.79 dose ratio) also resulted in statistically significant synergy (Fig. 3d). This same ratio (0.79) tested in additional clinical strains also resulted in broadly synergistic interactions (Fig. 4). In summary, IDentif.AI pinpointed PB/RA combination independently without any prior or existing information and importantly, the results aligned well with previous findings. With the emergence of new isolates and isolate variations across different regions, IDentif.AI can be engaged for a broad application across multiple isolates at reduced cost. A clinical trial that prospectively assesses IDentif.AI-pinpointed combinations may reveal further insights into the potential clinical application of combinations such as PB/RA. To further validate the potential of IDentif.AI as a sustainable approach in addressing AMR, IDentif.AI may be deployed to address other “ESKAPE” pathogens as part of future work.

### IDentif.AI-AMR: an alternate strategy against AMR

From 2019 to 2020, the United States alone has seen an estimated increase of 15% in AMR which is further aggravated by multi-drug resistance^48^. Therefore, new strategies must constantly evolve to keep pace with the increasing rate of AMR in this ongoing arms race. With only 91 prospectively assessed drug combinations, IDentif.AI was able to effectively pinpoint combinations including PB/RA and SAM/FDC as the top combinations against *A. baumannii*. The PB/RA combination was also determined by existing approaches, such as big data metabolomics^43^ and PK/PD modeling^44,49^. In a study that aimed to address *Klebsiella pneumoniae*, the PB/RA combination was paired with MEM in a triple-combination therapy^50^. PB/RA may potentially serve as the backbone of combinatorial designs in the space of AMR. Similarly, in a previous work, SAM/FDC was validated in vivo for *A. baumannii* treatment, and the results suggested that the combination may prevent the development of resistance during treatment^51^. These studies align well with the IDentif.AI results, which further support the feasibility of IDentif.AI in addressing AMR in *A. baumannii*.

Aside from IDentif.AI, other existing drug optimization approaches including stochastic search algorithm and statistical metamodeling have also provided insightful information on designing drug combinations. For example, in a study by Wong et al., a closed loop optimization modality guided by stochastic search algorithm was able to determine potent drug combinations against vesicular stomatitis virus in tens of iterations as compared to hundred thousand trials^52^. In another study, statistical metamodeling was applied to detect synergistic antimicrobial interactions. Specifically, drug cocktails were identified through factorial screening and response surface regression, which pointed to synergistic drug combinations^53^. IDentif.AI substantially differs from these platforms in that clinically relevant drug concentrations were utilized, ensuring the all drug-drug interactions are clinically feasible. More importantly, as opposed to performing factorial screening and response surface analysis separately, IDentif.AI utilizes OACD to efficiently estimate the linear, bilinear, and quadratic coefficients, enabling flash optimization of synergistic combinations. IDentif.AI-detected combinations with drug-drug interactions were initially assessed via response surfaces in Fig. 2 and subsequently, analyzed using Bliss independence model. Nonetheless, these approaches together may help contribute to future pandemic readiness and sustainably accelerate drug combination design against the ESKAPE pathogens.

### Mechanism-agnostic approach to identify synergistic drug interactions

Synergistic interactions are vital for drug development and combinations in antimicrobial therapy. β-lactams and β-lactamase inhibitors are commonly co-administered to act synergistically, restoring and enhancing the antimicrobial efficacy of β-lactams. They are found in many regimens, such as MEM/vaborbactam, ceftolozane/tazobactam, and ceftazidime/avibactam^54,55^. Mechanistically, β-lactamase inhibitors inhibit the activity of bacterial β-lactamases, which prevent β-lactam hydrolysis and weaken resistance. Consequently, the weakened resistance enables β-lactam to disrupt the cell wall synthesis process, and ultimately leading to bactericide. However, *A. baumannii* may be resistant due to carbapenemases (e.g. metallo-β-lactamases or OXA-type carbapenemases) not inhibited by the aforementioned novel beta-lactamase inhibitors, and is also known to develop resistance through non-enzymatic mechanisms such as activation of efflux pump, decreased in membrane permeability, or single point mutations^56,57^. As a result, existing regimens such as imipenem/relebactam and ceftazidime/avibactam, which were developed for carbapenemase-producing *Enterobacterales,* unfortunately do not possess adequate activity against *A. baumannii*, necessitating the discovery of novel agents or synergistic drug combinations in this arms race against AMR^58,59^. While novel agents such as sulbactam/durlobactam and cefepime/zidebactam are welcome developments, these are not widely available or not approved yet in various jurisdictions, and the development of resistance to these new agents is somewhat inevitable. Hence, testing as described in our study still has a role to play in discovering potential new therapeutic combinations, even with these new agents.

Without requiring any novel drug discovery which often involves a large number of limited healthcare resources, the mechanism-agnostic IDentif.AI platform can rapidly determine synergistic interactions and pinpoint the optimal drug combinations from an existing pool of FDA-approved drugs for sustainability. IDentif.AI has significant potential implications in improving sustainability for AMR treatment development by repurposing existing drugs in a short amount of time^13^. Instead of an empirical approach, IDentif.AI managed to pinpoint a broadly actionable and strongly synergistic PB/RA combination. PB/RA demonstrated concentration-dependent synergy, implying the necessity of calibrating drug doses for desired interplay and enhanced efficacy, which is also confirmed by clinical personalized medicine practices^60^. The synergistic effect was initially driven by PB that disrupts the lipopolysaccharide within the outer membrane, causes structural instability, and increases permeability, followed by RA inhibition of RNA synthesis^43^. With IDentif.AI’s ability to pinpoint optimal regimens, a more sustainable approach can be taken to narrow down relevant mechanistic studies to better understand the bacteria and their interactions with these drug combinations and aid in existing drug development strategies.

### The intersection of clinician-guided and AI-assisted drug selections

The very first step of the IDentif.AI workflow involved active and frequent engagement with clinicians and infectious disease experts at NCID in Singapore, and this process enabled helpful discussions that led to the selection of drugs that are clinically relevant and feasible (Fig. 1). This engagement ensures that IDentif.AI-pinpointed combinations are clinically actionable and potentially useful to the clinical team by diversifying their existing reservoir of treatment strategies. Bridging clinician-guided drug selection with the emergence of generative AI (GAI), such as ChatGPT developed by OpenAI, may potentially facilitate the process of comprehensively identifying actionable drug candidates. For example, a recent study reported the application of ChatGPT in medication therapy management, which successfully provided drug recommendations validated by clinical pharmacists^61^. Moreover, GAI has also been employed to prioritize repurposed drug candidates for Alzheimer’s disease^62^. The application of GAI in medicine may be further expanded to many other areas, such as drug discovery, drug development, and treatment strategies^62–66^, and may integrate with existing clinical workflow to make healthcare more efficient and accessible^67^.

### Study limitations

This study provided important insights into the design of combination therapies that may effectively inhibit the growth profiles of *A. baumannii*. Notably, IDentif.AI-pinpointed PB/RA combination demonstrated broadly applicable activity against four different clinical isolates. It is important to note that PB/RA was only optimized in *A. baumannii* {2023496441} and though it was broadly effective in other clinical isolates, the bacterial cultures did not respond to the combination uniformly. For example, PB/RA exhibited stronger synergistic interactions at lower concentrations (<L2) in C1718-B, while the combination exhibited interactions only at higher concentrations for the other isolates (Figs. 3 and 4). This may be attributed to the heterogeneity of the four clinical isolates. Future evaluations of factors including genetic variability and resistance mechanisms may help explain the observed differences across all isolates. Moreover, the cytotoxicity of IDentif.AI-pinpointed combinations was not evaluated in this study. Prior to potential clinical translation, the toxicity and tolerance profiles of these combinations should be carefully considered and assessed in subsequent preclinical and clinical studies. In addition, the dose-dependent synergistic interactions observed in PB/RA may suggest that in downstream preclinical and clinical studies, the dose ratio of the combination may need to be properly adjusted in order to achieve the optimal outcomes (Figs. 3 and 4). Furthermore, the bacterial cultures in this study were incubated at 35 °C, which is within the suggested range. However, to further enhance clinical relevance of findings from in vitro studies, future IDentif.AI studies should carefully consider all aspects of experimental design including incubation temperature (e.g. 37 °C).

In this study, IDentif.AI utilizes an OACD that evaluated three different concentration levels (L0, L1, and L2). However, some drug-drug interactions may be observed in higher or lower concentrations. For example, in Figs. 3 and 4, the drug combinations exhibited dose-dependent interactions at various different dose ratios. Therefore, evaluating only three concentration levels may limit the interrogation of other potential drug-drug interactions in the parameter space. In future studies, an OACD consisting of more concentration levels may help accentuate more potential drug-drug interactions. Moreover, though PB/RA demonstrated broadly applicable activity across four different clinical isolates, further evaluating other region-specific isolates of *A. baumannii* may provide more insightful information pertaining to the potential applicability of the combination. In addition, IDentif.AI harnessed experimentally generated data of 91 OACD combinations to sustainably interrogate the parameter space of 19,683 combinations. In a scenario where only 3-drug combinations are prioritized, screening all three-way combinations via approaches like high throughput screening may also serve as an alternative approach to rapidly pinpoint actionable combinations within a few days. However, these approaches may leverage more resources compared to IDentif.AI, which only required to screen 91 combinations. Nonetheless, integrating IDentif.AI with other drug screening approaches may further reduce the resources needed to sustainably and rapidly determine actionable drug combinations for future pandemic readiness. The efficacy of monotherapies and drug combinations were determined using %Inhibition. This, however, led to some %Inhibition results outside of the 0–100 range. For example, the RA/AN combination was shown to have −21.00 ± 15.37 %Inhibition. Aside from variations including well-to-well subtle differences, this may be attributed to “hormesis,” which is the adaptive response of bacteria to low-dose stimulation, or stress^39,68,69^. Similarly, low-dose drug interventions as seen in Supplementary Figs. 1 and 2 resulted in negative %Inhibition values, suggesting that hormetic effects may be more prevalent in low concentrations. Additionally, antibiotics alone and in combinations as well as the bacterial cultures consisting of different compounds may induce hormesis in bacterial growth^39,69,70^.

Nonetheless, this study presented a sustainable approach to accelerate the development of AMR-specific drug combinations and treatment strategies. This study also demonstrated a community-driven workflow that engages with clinicians and experts from NCID in Singapore to develop drug combinations against *A. baumannii*. Therefore, this workflow may be deployed to properly design drug combinations in the context of future pandemic readiness, regionally and internationally. In this study, IDentif.AI pinpointed PB/RA combination independently, which has also been studied in previous papers. Importantly, this combination along with others was experimentally derived with data acquired only in this study and did not rely on existing works. These results validate the actionability of IDentif.AI to locate effective drug combinations. Though IDentif.AI is a mechanism-agnostic platform, downstream in-depth mechanistic studies including PK/PD modeling may help provide further insights to explain the interactions of combinations presented in this study. In the future, broadening the drug pool to include off-labeled drugs may uncover unforeseen novel efficacious combinations for *A. baumannii*, further diversifying available treatment options for *A. baumannii* and its strains more sustainably.

## Methods

### Drugs and experimental model

Nine FDA-approved drugs of meropenem (MEM), tigecycline (TGC), polymyxin B (PB), minocycline (MI), amikacin (AN), ampicillin-sulbactam (SAM), rifampicin (RA), eravacycline (ERV), and cefiderocol (FDC) were purchased from Selleckchem (MEM: S1381, PB: S1395, TGC: S1403, MI: S4226), TargetMol (ERV: T11227, RA: T0681, AMP: T0814L, SUL: T1631, AN: T1013), and BOC Sciences (FDC: B0084-475177). FDC, ERV, and RA were dissolved in DMSO, while the rest were dissolved in water.

All experiments were conducted in a BSL-2 laboratory. A total of four *A. baumannii* clinical isolates (2023496441, 2033643894, C1687, and C1718-B) were tested in this study, and their susceptibility profiles were summarized in Supplementary Table 2. The *A. baumannii* {2023496441} and {2033643894} strains are clinical isolates in Singapore, which were sequenced in a previous study^71^. *A. baumannii* was cultured on blood agar plates (Thermo Scientific) overnight and resuspended in Mueller Hinton (MH) Cation adjusted broth (Becton Dickinson) to achieve a final density of 1 × 10^5^ CFU/mL. The bacterial cultures were incubated in 96-well microplates at 35 °C for 16–20 h without shaking and their growth profiles in the presence of antibiotic were measured by light absorbance (optical density; OD) at 600 nm (Multiskan FC) after brief shaking to ensure an even suspension. The %Inhibition is obtained by normalizing the OD_600_ of drug-free controls and treated bacterial cultures (Eq. 1).

1 $$\% {Inhibition}=\left(1-\frac{{Drug\; Treatment}}{{Drug\; Free\; Control}}\right)\times 100 \%$$

For the dose-response curves, each of the nine drugs was serially diluted (2-fold) into 12 different concentrations. The %Inhibition of each concentration was obtained via OD_600_ measurements of the cultures (*n* = 3). The dose-response curves were constructed by plotting the logarithmic scale of the concentrations and their corresponding %Inhibition values (GraphPad Prism 10; GraphPad Software). The IC_10_, IC_20_, and IC_50_ values were extracted from the dose–response curves.

### IDentif.AI analysis

A curated list of 91 strategic combinations in accordance with the OACD was experimentally validated (Supplementary Table 3). The design consists of 64 two-level fractional factorials and 27 three-level orthogonal arrays^72^. These combinations represent the number of combinations required to efficiently estimate the linear, bilinear, and quadratic coefficients for the second-order quadratic series. The experimentally derived %Inhibitions of all OACD combinations were determined from the OD_600_ from bacterial cultures exposed to the combinations (*n* = 3). IDentif.AI performed a stepwise regression and correlated the OACD combinations and their corresponding %Inhibitions via a second-order quadratic series, which was used to predict the %Inhibitions of the parameter space consisting of nine drugs in three concentration levels (3^9^ = 19,683) (MATLAB R2020b; MathWorks, Inc.) (Supplementary Table 4)^24,26–28^. Box-Cox transformation was performed to determine potential transformations that may improve the distribution of residuals and the goodness-of-fit of the IDentif.AI analysis (adjusted *R*^2^), and residual-based outlier analysis was also performed. Based on the ranked list, top and low-ranked combinations were pinpointed by IDentif.AI. Example IDentif.AI code is included in Supplementary Information. IDentif.AI-pinpointed top and low-ranked combinations were subsequently evaluated in vitro to confirm their efficacy against *A. baumannii* (*n* = 3). The experimentally derived %Inhibition data for validated IDentif.AI-pinpointed combinations were further compared with predicted data via Pearson correlation coefficient and other relevant analyses (Supplementary Fig. 4).

### Checkerboard synergy analysis

The two combinations pointed out by IDentif.AI (SAM/FDC and PB/RA) were further assessed via a 7 × 7 checkerboard assay. The highest concentrations of all drugs were prepared at 4x of their original L2 concentrations, and they were subsequently prepared in twofold dilutions. SAM and FDC concentration ranges were 4.694–150.160 μg/mL and 0.150–4.784 μg/mL, respectively. PB and RA concentration ranges were 0.0691–2.212 μg/mL and 0.0875–2.800 μg/mL, respectively. The *A. baumannii* bacterial cultures were exposed to drug combinations at various concentration ratios, following the same protocol previously described. The %Inhibition values were obtained and fed into GraphPad Prism 10 (GraphPad Software) to construct an interaction map for each checkerboard. Subsequently, the dataset was uploaded to SynergyFinder+ to quantify synergy at each dose ratio using Bliss independence model^26–28,40,41^. Resulting synergy scores for each dose ratio across the entire 7 × 7 checkerboard were fed into GraphPad Prism 10 to generate synergy maps (GraphPad Software).

### Statistical analysis

All in vitro experiments were performed in three biological replicates (*n* = 3). Data are presented in mean ± propagated SD (*n* = 3)^12,24,26,73^. IDentif.AI analysis was performed using the built-in function of stepwise regression in MATLAB R2020b (MathWorks, Inc.). The IDentif.AI-estimated coefficients were analyzed using the sum of square *F*-test, and the *P*-values of the estimates served as the exclusion criteria for stepwise regression. The distribution of experimental data was assessed using Shapiro-Wilk normality test. The significance of Bliss δ-synergy scores was assessed using one-sample *t*-test (*P < 0.05, **P < 0.01, and ***P < 0.001). The statistical significance of IDentif.AI-pinpointed combinations was determined using Kruskal Wallis test and Dunn’s post hoc test (*P < 0.05). The assay quality was assessed using Z’-factor, which is based on the separation of blank and drug free controls^74^. For %Inhibition calculations, aside from hormetic effects, variations arising from controls (drug-free and blank) and well-to-well subtle differences (e.g. temperature, oxygen level, and metabolism) may also result in %Inhibitions outside of the normal 0–100 range (e.g. <0 and >100).

## Supplementary information

Supplementary Information

Supplementary Data

## Data Availability

All data can be found in the manuscript or in Supplementary Information. The Supplementary Data includes all raw data of the dose-response curves and the 91 OACD combinations.

## Code availability

An example IDentif.AI code (MATLAB) is in Supplementary Information.
