# Supplementary material for: Flash optimization of drug combinations for Acinetobacter baumannii with IDentif.AI-AMR
Source: NPJ Antimicrob Resist. 2025 Feb 21;3:12. doi: 10.1038/s44259-025-00079-2 (PMC11845484; doi:10.1038/s44259-025-00079-2)
Supplement: Supplementary file 1 — Supplementary Information [file 44259_2025_79_MOESM1_ESM.docx]

**Flash optimization of drug combinations for *Acinetobacter baumannii* with IDentif.AI-AMR**

# Kui You, Yazid Nurhidayah Binte Mohamed, Li Ming Chong, Lissa Hooi, Peter Wang, Isaiah Zhe'En Zhuang, Stephen Chua, Ethan Lim, Alrick Zi Xin Kok, Kalisvar Marimuthu*, Shawn Vasoo*, Oon Tek Ng*, Conrad E.Z. Chan*, Edward Kai-Hua Chow*, Dean Ho*

**This Supplementary Information includes:**

- Supplementary Tables 1-4
- Supplementary Figs. 1-4
- Example Code
- References

**Supplementary Table 1 | Details of the selected nine drugs for IDentif.AI analysis.** The mechanism of action and minimum inhibitory concentration (MIC) resistance breakpoint for each drug are summarized below.

|  | **Mechanism of Action** | **Breakpoint for Resistance** |
| --- | --- | --- |
| Amikacin | Interfere with bacterial protein synthesis^1^ | >8 mg/L^2^ |
| Ampicillin-sulbactam | Ampicillin disrupts the cell wall formation of bacteria and inhibits β-lactamase for the sulbactam activity^3^ | - |
| Cefiderocol | Its chlorocatechol group allows it to easily cross bacterial membrane and accumulate inside to exert activity^4^ | >16 mg/L^5^ |
| Eravacycline | Bind to bacterial ribosomes and inhibits protein synthesis^6^ | - |
| Meropenem | Disrupt bacterial cell wall formation, leading to bacterial death^7^ | >8 mg/L^2^ |
| Minocycline | Bind to bacterial ribosomes and inhibits protein synthesis^8^ | >16 mg/L^9^ |
| Polymyxin B | Target and disrupt bacterial membrane^10^ | >4 mg/L^11^ |
| Rifampicin | Target DNA-dependent RNA polymerase to prevent bacterial RNA synthesis^12^ | >4 mg/L^13^ |
| Tigecycline | Block entry of transfer RNA by binding to bacterial ribosomes^14^ | - |

**Supplementary Table 2 | Resistance profiles of four tested *A. baumannii* isolates.** Susceptibility of four *A. baumannii* isolates {2023496441, 2033643894, C1687, and C1718-B} to common antimicrobials is tabulated below. R: resistant, I: intermediate, S: sensitive.

|  | **2023496441** | **2033643894** | **C1687** | **C1718-B** |
| --- | --- | --- | --- | --- |
| Amikacin | R | R | R | R |
| Ampicillin-sulbactam | R | R | R | S |
| Cefepime | R | R | R | R |
| Ceftazidime | R | R | R | R |
| Ceftriaxone | R | R | R | R |
| Ciprofloxacin | R | R | R | R |
| Gentamicin | R | R | R | R |
| Meropenem | R | R | R | R |
| Minocycline | I | I | - | - |
| Piperacillin-tazobactam | R | R | R | R |
| Polymyxin B | I | I | - | - |
| Trimethoprim-sulfamethoxazole | R | R | S | S |

**Supplementary Table 3 | 91-combination resolution IV OACD.** -1, 0, and 1 in the table correspond to level 0 (L0), L1, and L2 drug concentrations, respectively. Amikacin (AN), ampicillin-sulbactam (AMP-SUL), cefiderocol (FDC), eravacycline (ERV), meropenem (MEM), minocycline (MI), polymyxin B (PXM), rifampicin (RA), tigecycline (TGC).

|  | **AN** | **AMP-SUL** | **FDC** | **ERV** | **MEM** | **MI** | **PB** | **RA** | **TGC** |
| --- | --- | --- | --- | --- | --- | --- | --- | --- | --- |
| 1 | -1 | -1 | -1 | -1 | -1 | -1 | -1 | 1 | -1 |
| 2 | 1 | -1 | -1 | -1 | -1 | -1 | 1 | -1 | 1 |
| 3 | -1 | 1 | -1 | -1 | -1 | -1 | 1 | -1 | -1 |
| 4 | 1 | 1 | -1 | -1 | -1 | -1 | -1 | 1 | 1 |
| 5 | -1 | -1 | 1 | -1 | -1 | -1 | 1 | -1 | -1 |
| 6 | 1 | -1 | 1 | -1 | -1 | -1 | -1 | 1 | 1 |
| 7 | -1 | 1 | 1 | -1 | -1 | -1 | -1 | 1 | -1 |
| 8 | 1 | 1 | 1 | -1 | -1 | -1 | 1 | -1 | 1 |
| 9 | -1 | -1 | -1 | 1 | -1 | -1 | 1 | 1 | 1 |
| 10 | 1 | -1 | -1 | 1 | -1 | -1 | -1 | -1 | -1 |
| 11 | -1 | 1 | -1 | 1 | -1 | -1 | -1 | -1 | 1 |
| 12 | 1 | 1 | -1 | 1 | -1 | -1 | 1 | 1 | -1 |
| 13 | -1 | -1 | 1 | 1 | -1 | -1 | -1 | -1 | 1 |
| 14 | 1 | -1 | 1 | 1 | -1 | -1 | 1 | 1 | -1 |
| 15 | -1 | 1 | 1 | 1 | -1 | -1 | 1 | 1 | 1 |
| 16 | 1 | 1 | 1 | 1 | -1 | -1 | -1 | -1 | -1 |
| 17 | -1 | -1 | -1 | -1 | 1 | -1 | 1 | 1 | -1 |
| 18 | 1 | -1 | -1 | -1 | 1 | -1 | -1 | -1 | 1 |
| 19 | -1 | 1 | -1 | -1 | 1 | -1 | -1 | -1 | -1 |
| 20 | 1 | 1 | -1 | -1 | 1 | -1 | 1 | 1 | 1 |
| 21 | -1 | -1 | 1 | -1 | 1 | -1 | -1 | -1 | -1 |
| 22 | 1 | -1 | 1 | -1 | 1 | -1 | 1 | 1 | 1 |
| 23 | -1 | 1 | 1 | -1 | 1 | -1 | 1 | 1 | -1 |
| 24 | 1 | 1 | 1 | -1 | 1 | -1 | -1 | -1 | 1 |
| 25 | -1 | -1 | -1 | 1 | 1 | -1 | -1 | 1 | 1 |
| 26 | 1 | -1 | -1 | 1 | 1 | -1 | 1 | -1 | -1 |
| 27 | -1 | 1 | -1 | 1 | 1 | -1 | 1 | -1 | 1 |
| 28 | 1 | 1 | -1 | 1 | 1 | -1 | -1 | 1 | -1 |
| 29 | -1 | -1 | 1 | 1 | 1 | -1 | 1 | -1 | 1 |
| 30 | 1 | -1 | 1 | 1 | 1 | -1 | -1 | 1 | -1 |
| 31 | -1 | 1 | 1 | 1 | 1 | -1 | -1 | 1 | 1 |
| 32 | 1 | 1 | 1 | 1 | 1 | -1 | 1 | -1 | -1 |
| 33 | -1 | -1 | -1 | -1 | -1 | 1 | -1 | -1 | 1 |
| 34 | 1 | -1 | -1 | -1 | -1 | 1 | 1 | 1 | -1 |
| 35 | -1 | 1 | -1 | -1 | -1 | 1 | 1 | 1 | 1 |
| 36 | 1 | 1 | -1 | -1 | -1 | 1 | -1 | -1 | -1 |
| 37 | -1 | -1 | 1 | -1 | -1 | 1 | 1 | 1 | 1 |
| 38 | 1 | -1 | 1 | -1 | -1 | 1 | -1 | -1 | -1 |
| 39 | -1 | 1 | 1 | -1 | -1 | 1 | -1 | -1 | 1 |
| 40 | 1 | 1 | 1 | -1 | -1 | 1 | 1 | 1 | -1 |
| 41 | -1 | -1 | -1 | 1 | -1 | 1 | 1 | -1 | -1 |
| 42 | 1 | -1 | -1 | 1 | -1 | 1 | -1 | 1 | 1 |
| 43 | -1 | 1 | -1 | 1 | -1 | 1 | -1 | 1 | -1 |
| 44 | 1 | 1 | -1 | 1 | -1 | 1 | 1 | -1 | 1 |
| 45 | -1 | -1 | 1 | 1 | -1 | 1 | -1 | 1 | -1 |
| 46 | 1 | -1 | 1 | 1 | -1 | 1 | 1 | -1 | 1 |
| 47 | -1 | 1 | 1 | 1 | -1 | 1 | 1 | -1 | -1 |
| 48 | 1 | 1 | 1 | 1 | -1 | 1 | -1 | 1 | 1 |
| 49 | -1 | -1 | -1 | -1 | 1 | 1 | 1 | -1 | 1 |
| 50 | 1 | -1 | -1 | -1 | 1 | 1 | -1 | 1 | -1 |
| 51 | -1 | 1 | -1 | -1 | 1 | 1 | -1 | 1 | 1 |
| 52 | 1 | 1 | -1 | -1 | 1 | 1 | 1 | -1 | -1 |
| 53 | -1 | -1 | 1 | -1 | 1 | 1 | -1 | 1 | 1 |
| 54 | 1 | -1 | 1 | -1 | 1 | 1 | 1 | -1 | -1 |
| 55 | -1 | 1 | 1 | -1 | 1 | 1 | 1 | -1 | 1 |
| 56 | 1 | 1 | 1 | -1 | 1 | 1 | -1 | 1 | -1 |
| 57 | -1 | -1 | -1 | 1 | 1 | 1 | -1 | -1 | -1 |
| 58 | 1 | -1 | -1 | 1 | 1 | 1 | 1 | 1 | 1 |
| 59 | -1 | 1 | -1 | 1 | 1 | 1 | 1 | 1 | -1 |
| 60 | 1 | 1 | -1 | 1 | 1 | 1 | -1 | -1 | 1 |
| 61 | -1 | -1 | 1 | 1 | 1 | 1 | 1 | 1 | -1 |
| 62 | 1 | -1 | 1 | 1 | 1 | 1 | -1 | -1 | 1 |
| 63 | -1 | 1 | 1 | 1 | 1 | 1 | -1 | -1 | -1 |
| 64 | 1 | 1 | 1 | 1 | 1 | 1 | 1 | 1 | 1 |
| 65 | -1 | -1 | -1 | -1 | -1 | -1 | -1 | -1 | -1 |
| 66 | -1 | 0 | 1 | -1 | 1 | 0 | -1 | 0 | 1 |
| 67 | -1 | 1 | 0 | -1 | 0 | 1 | -1 | 1 | 0 |
| 68 | -1 | -1 | 0 | 0 | 0 | -1 | 1 | 0 | 1 |
| 69 | -1 | 0 | -1 | 0 | -1 | 0 | 1 | 1 | 0 |
| 70 | -1 | 1 | 1 | 0 | 1 | 1 | 1 | -1 | -1 |
| 71 | -1 | -1 | 1 | 1 | 1 | -1 | 0 | 1 | 0 |
| 72 | -1 | 0 | 0 | 1 | 0 | 0 | 0 | -1 | -1 |
| 73 | -1 | 1 | -1 | 1 | -1 | 1 | 0 | 0 | 1 |
| 74 | 0 | -1 | -1 | -1 | 0 | 0 | 0 | 0 | 0 |
| 75 | 0 | 0 | 1 | -1 | -1 | 1 | 0 | 1 | -1 |
| 76 | 0 | 1 | 0 | -1 | 1 | -1 | 0 | -1 | 1 |
| 77 | 0 | -1 | 0 | 0 | 1 | 0 | -1 | 1 | -1 |
| 78 | 0 | 0 | -1 | 0 | 0 | 1 | -1 | -1 | 1 |
| 79 | 0 | 1 | 1 | 0 | -1 | -1 | -1 | 0 | 0 |
| 80 | 0 | -1 | 1 | 1 | -1 | 0 | 1 | -1 | 1 |
| 81 | 0 | 0 | 0 | 1 | 1 | 1 | 1 | 0 | 0 |
| 82 | 0 | 1 | -1 | 1 | 0 | -1 | 1 | 1 | -1 |
| 83 | 1 | -1 | -1 | -1 | 1 | 1 | 1 | 1 | 1 |
| 84 | 1 | 0 | 1 | -1 | 0 | -1 | 1 | -1 | 0 |
| 85 | 1 | 1 | 0 | -1 | -1 | 0 | 1 | 0 | -1 |
| 86 | 1 | -1 | 0 | 0 | -1 | 1 | 0 | -1 | 0 |
| 87 | 1 | 0 | -1 | 0 | 1 | -1 | 0 | 0 | -1 |
| 88 | 1 | 1 | 1 | 0 | 0 | 0 | 0 | 1 | 1 |
| 89 | 1 | -1 | 1 | 1 | 0 | 1 | -1 | 0 | -1 |
| 90 | 1 | 0 | 0 | 1 | -1 | -1 | -1 | 1 | 1 |
| 91 | 1 | 1 | -1 | 1 | 1 | 0 | -1 | -1 | 0 |

**Supplementary Table 4 | IDentif.AI-estimated coefficients for the interrogation of the %Inhibition data.** No transformation was applied to the %Inhibition data. Statistical significance was determined using *F*-test. Amikacin (AN), ampicillin-sulbactam (AMP-SUL), cefiderocol (FDC), eravacycline (ERV), meropenem (MEM), minocycline (MI), polymyxin B (PXM), rifampicin (RA), tigecycline (TGC).

| **Term** | **Estimate** | **SE** | ***t*-statistic** | ***p*-value** |
| --- | --- | --- | --- | --- |
| Intercept | 84.359 | 5.834 | 14.459 | 3.06 × 10^-36^ |
| AN | -0.502 | 1.311 | -0.383 | 0.70 |
| AMP-SUL | 10.286 | 1.308 | 7.865 | 6.91 × 10^-14^ |
| FDC | 22.402 | 1.316 | 17.018 | 8.32 × 10^-46^ |
| ERV | -0.549 | 1.303 | -0.421 | 0.67 |
| MEM | -0.093 | 1.324 | -0.070 | 0.94 |
| MI | 3.824 | 1.316 | 2.907 | 3.93 × 10^-3^ |
| PB | 6.154 | 1.301 | 4.729 | 3.49 × 10^-6^ |
| RA | 11.891 | 1.302 | 9.133 | 1.06 × 10^-17^ |
| TGC | 2.788 | 1.308 | 2.131 | 0.03 |
| AN:AMP-SUL | -3.071 | 1.357 | -2.262 | 0.02 |
| AN:FDC | 2.663 | 1.351 | 1.971 | 0.05 |
| AN:MEM | -2.521 | 1.356 | -1.859 | 0.06 |
| AMP-SUL:MI | -4.006 | 1.360 | -2.946 | 3.48 × 10^-3^ |
| AMP-SBT:PMB | -5.565 | 1.349 | -4.126 | 4.80 × 10^-5^ |
| AMX-SUL:RA | -2.546 | 1.344 | -1.895 | 0.06 |
| AMP-SUL:TGC | -3.233 | 1.356 | -2.383 | 0.02 |
| FDC:MEM | -3.336 | 1.357 | -2.458 | 0.01 |
| ERV:MI | -3.138 | 1.351 | -2.323 | 0.02 |
| ERV:RA | 2.293 | 1.358 | 1.689 | 0.09 |
| ERV:TGC | -5.292 | 1.345 | -3.935 | 1.04 × 10^-4^ |
| MEM:MI | -2.608 | 1.332 | -1.957 | 0.05 |
| MEM:TGC | -2.253 | 1.356 | -1.662 | 0.10 |
| MI:TGC | -2.669 | 1.355 | -1.970 | 0.05 |
| PB:RMP | 4.278 | 1.355 | 3.156 | 2.00 × 10^-3^ |
| PB:TGC | -3.435 | 1.347 | -2.550 | 0.01 |
| AN^2^ | -7.593 | 4.364 | -1.740 | 0.08 |
| FDC^2^ | -8.645 | 4.382 | -1.973 | 0.05 |
| ERV^2^ | 7.882 | 4.346 | 1.813 | 0.07 |
| MI^2^ | -10.876 | 4.376 | -2.485 | 0.01 |
| **Model Statistics** | | | | |
| Number of Observations | | | 327 | |
| Error Degrees of Freedom | | | 297 | |
| Root Mean Squared Error | | | 21.2 | |
| R^2^ | | | 0.788 | |
| Adjusted R^2^ | | | 0.767 | |
| *F*-statistic vs. Constant Model | | | 38 | |
| *p*-value | | | 8.91 × 10^-83^ | |
| Fitting Correlation | | | 0.887 | |

**Supplementary Fig. 1 | Dose response analysis.** All selected nine drugs were serial diluted into 12 concentrations and their %Inhibitions were prospectively measured. The logarithmic scale of drug concentrations was plotted against the measured %Inhibitions. IC_50_ extracted from the dose response curves of each drug are labelled in the top left corner. Data are presented in mean ± propagated SD (n = 3).

**Supplementary Fig. 2 | Experimental results of OACD combinations and monotherapies. a** Prospectively measured %Inhibition of all 91 OACD combinations and **b** monotherapies of all nine drugs at L1 and L2 concentrations. Data are presented in mean ± propagated SD (n = 3). Amikacin (AN), ampicillin-sulbactam (AMP-SUL), cefiderocol (FDC), eravacycline (ERV), meropenem (MEM), minocycline (MI), polymyxin B (PXM), rifampicin (RA), tigecycline (TGC).

**Supplementary Fig. 3 | Residual analysis of IDentif.AI analysis on the %Inhibition data**. Distribution of residuals and Cook’s distance did not point to potential outliers, and histogram of residuals suggested that the data is normally distributed.


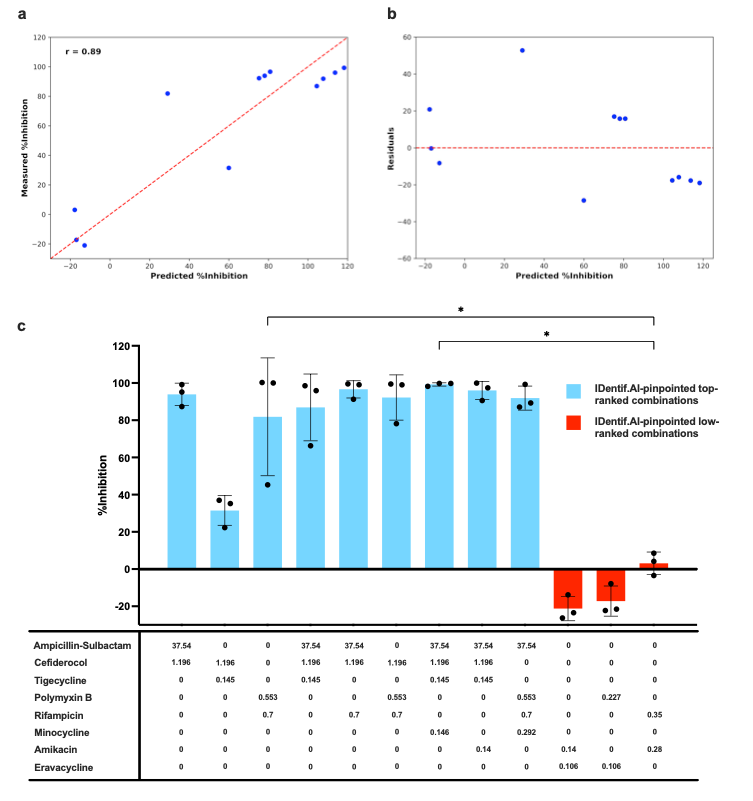


**Supplementary Fig. 4 | Validation of IDentif.AI-pinpointed top and low ranked combinations**. **a** The predicted %Inhibitions of shortlisted IDentif.AI-pinpointed combinations in the validation study were plotted against the experimentally derived efficacy data. The Pearson correlation coefficient (r) is 0.89. **b** The residuals of the above-mentioned combinations are also plotted. **c** The %Inhibitions of top and low ranked IDentif.AI-pinpointed combinations are plotted along with their respective drug concentrations (n = 3). Kruskal-Wallis test determined statistically significant difference among the combinations (*P* = 0.005), followed by Dunn’s post hoc pairwise comparisons (**P* < 0.05).

**Example Code**

%Load OACD Data and Corresponding %Inhibitions

data =

[

| MEM | TGC | PB | MI | AN | SAM | RA | ERV | FDC | %Inhibition |
| --- | --- | --- | --- | --- | --- | --- | --- | --- | --- |
| -1 | -1 | -1 | -1 | -1 | -1 | -1 | 1 | -1 | 0.54 |
| 1 | -1 | -1 | -1 | -1 | -1 | 1 | -1 | 1 | 24.29 |
| -1 | 1 | -1 | -1 | -1 | -1 | 1 | -1 | -1 | 32.42 |
| 1 | 1 | -1 | -1 | -1 | -1 | -1 | 1 | 1 | 65.76 |
| -1 | -1 | 1 | -1 | -1 | -1 | 1 | -1 | -1 | 74.17 |
| 1 | -1 | 1 | -1 | -1 | -1 | -1 | 1 | 1 | 100.57 |
| -1 | 1 | 1 | -1 | -1 | -1 | -1 | 1 | -1 | 100.67 |
| 1 | 1 | 1 | -1 | -1 | -1 | 1 | -1 | 1 | 100.02 |
| -1 | -1 | -1 | -1 | -1 | -1 | -1 | 1 | -1 | 60.67 |

]

%Define Inputs and Outputs

x = data(: , 1:9);

y = data(: , 10);

%IDentif.AI Quadratic Series

result = stepwiselm(x , y, ‘quadratic’, 'ResponseVar', 'Inhibition', 'PredictorVars', {‘MEM’, ‘TGC’, ‘PB’, ‘MI’, ‘AN’, ‘SAM’, ‘RA’, ‘ERV’, ‘FDC’});

**References**

1. Sizar, O., Rahman, S. & Sundareshan, V. Amikacin. in *StatPearls* (StatPearls Publishing, Treasure Island (FL), 2024).

2. EUCAST. Breakpoint tables for interpretation of MICs and zone diameters. Version 14.0. (2024).

3. Peechakara, B. V. & Gupta, M. Ampicillin/Sulbactam. in *StatPearls* (StatPearls Publishing, Treasure Island (FL), 2024).

4. Soriano, A. & Mensa, J. Mechanism of action of cefiderocol. *Rev Esp Quimioter* **35**, 16–19 (2022).

5. CLSI. Performance Standards for Antimicrobial Susceptibility Testing. 33rd ed. CLSI supplement M100. (2023).

6. Eravacycline. in *LiverTox: Clinical and Research Information on Drug-Induced Liver Injury* (National Institute of Diabetes and Digestive and Kidney Diseases, Bethesda (MD), 2012).

7. Dhillon, S. Meropenem/Vaborbactam: A Review in Complicated Urinary Tract Infections. *Drugs* **78**, 1259–1270 (2018).

8. Asadi, A. *et al.* Minocycline, focus on mechanisms of resistance, antibacterial activity, and clinical effectiveness: Back to the future. *J. Glob. Antimicrob. Resist.* **22**, 161–174 (2020).

9. Tsakris, A., Koumaki, V. & Dokoumetzidis, A. Minocycline susceptibility breakpoints for Acinetobacter baumannii: do we need to re-evaluate them? *J. Antimicrob. Chemother.* **74**, 295–297 (2019).

10. Zavascki, A. P., Goldani, L. Z., Li, J. & Nation, R. L. Polymyxin B for the treatment of multidrug-resistant pathogens: a critical review. *J. Antimicrob. Chemother.* **60**, 1206–1215 (2007).

11. Research, C. for D. E. and. FDA Rationale for Polymyxin Breakpoints for Enterobacterales, Pseudomonas aeruginosa, and Acinetobacter spp. *FDA* (2023).

12. Beloor Suresh, A., Rosani, A., Patel, P. & Wadhwa, R. Rifampin. in *StatPearls* (StatPearls Publishing, Treasure Island (FL), 2024).

13. Nwabor, L. C. *et al.* Genotypic and phenotypic mechanisms underlying antimicrobial resistance and synergistic efficacy of rifampicin-based combinations against carbapenem-resistant *Acinetobacter baumannii*. *Heliyon* **10**, e27326; 10.1016/j.heliyon.2024.e27326 (2024).

14. Greer, N. D. Tigecycline (Tygacil): the first in the glycylcycline class of antibiotics. *Proc (Bayl Univ Med Cent)* **19**, 155–161 (2006).
